# Report of the Water Commissioners of Boston on the Material Best Adapted for Distribution Water Pipes; and on the Most Economical Mode of Introducing Water into Private Houses

**Published:** 1848-10

**Authors:** 


*Report of the Water Commissioners on the Material best adapted for Distribution Water Pipes; and on the most Economical Mode of Introducing Water into Private Houses.* 8vo. pp. 67. Boston, 1848.

This—like the last work noticed—is a municipal production. It is a well executed Report, mainly as to the propriety of employing leaden service pipes in the introduction of the water of the Cochituate Lake into Boston; and its conclusions appear to us as unanswerable as the positions and arguments are soundly logical. "Upon a careful examination of this mass of testimony," say the Water Commissioners—Messrs. Nathan Hale and Thomas B. Curtis—"we regard it as satisfactorily proved, that the water of Cochituate Lake, which is about to be introduced into the city [Boston], may be safely distributed to private dwellings, by means of leaden pipes, without danger to the health of those who may freely use it with their food." p. 19.

Much excitement, it appears, has prevailed in Boston on this question, arising from the fact, that the water of certain wells does act rapidly on lead exposed to it; and hence it became important to see what would be the effect of the water of the lake on that metal. This was shown to be analogous to that observed in the case of the waters of the Thames, Schuylkill, Croton, &c., being in no case to such a degree as to act injuriously on the system. The Board of Consulting Physicians of the City not having, in their Report to the City Council specified or expressed an opinion as to the material most entitled to preference for water pipes, the Water Commissioners, in coming to a decision, paid "careful attention to the information and opinions of the scientific gentlemen who have given replies to the inquiries addressed to them by the Board of Consulting Physicians, and particularly the results of the very thorough investigation and experiments of Professor Horsford, of Harvard University." "These results"—they properly remark—"appear to us to be of great value, and in corroboration of the great mass of evidence derived from a very extensive observation of the use of leaden pipes for the supply of cities and towns, for a long series of years, entirely satisfactory and conclusive."

We extract—although at considerable length—the opinion of the Commissioners on the subject of the best material for water pipes, and on the interesting results arrived at by Professor Horsford.

"While this subject was undergoing the investigation of the consulting physicians, and of the eminent chemists who had been invited to aid them in the inquiry, the Water Commissioners were under the necessity of beginning the work of laying down the distribution pipes. They deemed it improper to make use of a material which might in the result be proscribed, as dangerous to the health of the citizens. They accordingly procured iron pipes of one and a half and two inches in diameter, to be cast, which have been laid down for carrying the water from the street mains to the sidewalks, and in part to the dwelling houses, so far as this branch of the work has been yet accomplished.

"The cost of pipes of this description, including the laying down, is considerably higher than that of pipes of lead, independently of the cost of making additional joints, where they are required. There is also a further objection to the use of these pipes, that with the greatest caution which can be used in laying them, they are more liable to be broken than pipes of lead, or other flexible metal.

"In the mean time, we have given attention to experiments which have been made of pipes constructed of various other materials. Tin has been used for coating the internal surface of pipes of iron, lead and copper, for the purpose of preserving them against the action of the water upon those metals. Pipes of each of these descriptions have been strongly recommended, on some limited experience, but we are of opinion that there is not sufficient evidence of the durability of the coating, in either form, to justify its adoption for generál use. Pipes of block tin appear to be in some respects preferable to either description of those formed of other metals, and merely coated with tin. The cost of tin per pound is about four times that of lead, but as it is of greater tenacity than lead, a smaller quantity of metal serves to give the pipes a sufficient degree of strength, so that pipes composed of block tin, of a suitable thickness, can be procured at about double the cost of pipes of equal strength composed of lead. But the experiments detailed in the reports of Professor Horsford, as well as information derived from other sources, show that tin is gradually dissolved by the Cochituate and other similar waters; and that the decomposition does not in a short time cease, like the lead in the same water, but continues, as far as any experiment has been made, indefinitely. It is also liable to rapid decomposition, by being brought in contact externally with certain acids and gases, to which in various positions it will be exposed. Whether any sensible deleterious effect upon the water is produced by the gradual decomposition of the tin pipe, is a question which has not been satisfactorily determined. But for the reasons briefly stated, we are of opinion that, independently of the question of comparative cost, tin is no better adapted for the distribution of the water of Cochituate Lake than lead, and that probably it would prove less durable.

" Pipes manufactured of malleable iron are used to some extent in various places, for the distribution of water for domestic uses. They are in every respect well adapted to the purpose, with the exception of their liability to corrode, by the action of the water within, as well as the effects of moisture on the external surface. They are stronger than lead, and not more expensive. They can be made of any desirable dimensions, and are not liable like cast iron to be broken, by an unequal pressure on the different parts. The experience of their use, however, so far as it has come to our knowledge, is too limited to enable us to form a positive judgment of the force of the objection above mentioned. It has been apprehended, that the effect of rust would be such as to render the water unfit for use in the washing of clothes and linen, and in process of time, to close the aperture of the pipe.

" Pipes formed of sheet iron, coated internally with hydraulic cement, have been recently introduced, and they promise to be highly useful under certain circumstances. When laid in the earth, and and in situations exposing them externally to moisture, they are protected by a covering of hydraulic cement, which, besides preserving the iron against rust, gives an additional strength to the pipe. Whether they can be economically used for the distribution of water from the mains, has not been fully determined by any experiment within our knowledge.

" The consulting Physicians, in their report above referred to, although they did not recommend the use of distribution pipes composed of lead, strongly intimated the expectation that the doubts which they entertained might be removed by further experiments. It was important to reconcile the fact, that on immersing lead in water taken from the Fairmount, Croton and Jamaica Pond Water Works, it undergoes a perceptible partial dissolution, with the well attested evidence, that a large portion of the population of the cities of Philadelphia, New York and Boston, are in the constant use of water from those works, drawn through leaden pipes, without experiencing from it any injurious effects. The experiments which had been at that date begun by Professor Horsford, and have been since more thoroughly prosecuted by him, afford in our opinion a satisfactory solution of this apparent contradiction. These experiments demonstrate that the action of the comparatively pure water of lakes and rivers, upon bright bars of lead, which on their immersion in it, is distinctly perceptible, ceases after a period of a few days; and that this immediate action of the water, upon the surface of lead, forms a coating, which, for all practical purposes, is impervious to water, and entirely insoluble in it. This coating remains unchanged during any period in which it has thus far been immersed; its appearance after some months or years of immersion, in the case of the Croton, is quite the same, as within three or four days from the first immersion. The water on the first and second days in which the lead is so immersed, and during the continuance of any perceptible action on the surface of the leaden bars, shows traces of a mixture of lead, on trial by the ordinary tests; but on the repeated removal of this water, and substitution of other water from the same source, after the coating is formed, no trace of lead is discoverable by the most effective tests, after any length of exposure of the water in contact with the lead, which will ordinarily occur.

"It has, however, never been doubted by those who have investigated this subject, that the water of wells and springs of certain descriptions, and in certain situations, exerts a much more powerful and a continued effect upon lead with which it comes in contact; and that cases of paralysis, colic, and even death, have been traced to the drinking of water contaminated by this poisonous mixture. The negative evidence that no well authenticated cases of these diseases have occurred, in consequence of drinking the waters furnished by the public water works of the cities of London, Philadelphia, New York, and many other places, when distributed through leaden pipes, authorizes the belief, that the scattered cases of disease of these descriptions, which have been usually traced to the use of water from wells and springs, have arisen from some property peculiar to the water from those sources, and not common to water derived from lakes and rivers. Attempts have accordingly been made, to discover the nature and sources of the mixtures, which impart to water the power of acting more energetically upon lead. It is observed that nitrates possess this power, and that they are frequently found in well water. The observations of Professor Horsford have led him to the conclusion, that the unequal proportion of these salts constitutes the chief distinction between different waters, in their relation to lead. These salts are often, if not uniformly found, in the water of wells and springs, so situated as to be replenished by the filtration of water through a soil enriched from the stable, or by the wash from collections of animal substances of any description. A small solution of saltpetre, or of a nitrate of any description, in water, is found to impart to it the property of dissolving lead, and thereby forming the nitrate of lead. This substance renders the water undoubtedly deleterious, and dangerous to the health of those who drink it, or use it in the preparation of their food. This explanation, which seems to be fully confirmed by ample experiments, accounts sufficiently for the fact, that the water of wells situated, as are a large portion of those in town and cities, and of springs situated in the midst of richly cultivated fields, or in the vicinity of animal deposites of any description, may produce the chemical effect here described, upon the leaden pipes used to conduct it, while the waters of rivers and lakes, not particularly exposed to contact with substances of that nature will be destitute of any such power.

" So long as it remained unknown what ingredients imparted to water the property of acting upon the surface of a leaden pipe, in such manner as to convert it into an active poison, the fact that the water flowing from a particular source was harmless at one time, did not afford a satisfactory assurance against its becoming dangerous at another; especially when it was fully ascertained, that it possessed the property of dissolving lead in a sensible degree, on its first immersion in it. But since it has been discovered as the result of repeated trials, that the effect of the waters of the Schuylkill and Croton rivers, and of Cochituate and Jamaica lakes, upon lead, is limited to a short period from its first immersion, and that by this temporary effect, there is invariably produced an indissoluble coating on the surface of the lead, which permanently protects is against any further action of the water upon it, and consequently preserves the water against imbibing any poisonous property; and since it is further ascertained that the more efficient power of dissolving lead, which is found to reside in certain waters apparently pure, is imparted by a substance rarely if ever found, except in a very minute degree, in the water of lakes and rivers, but which is often found in the water of wells and springs, there appears to be no longer any good ground to apprehend injurious effects upon water, of the former description, from its being transmitted through leaden pipes. A perceptible line of distinction is thus drawn, between a class of waters which are liable to acquire the property of imbibing a poisonous substance, by contact with lead, and another class, which, in a very wide experience of their use for domestic purposes, have been found not to possess that property.

" For the evidence of these facts, we refer to the several reports of Professor Horsford, appended to the report of the Board of Consulting Physicians, and (until the publication of a more detailed report of his further experiments) to his letters subjoined to this report, and to the corroborative documents annexed.

" Professor Horsford, in the letter dated July 25th, expresses the following opinion: 'Without an attempt at further enumeration of the conclusions at which I have arrived, I may state, with whatever of emphasis uninterrupted investigation from the first of last February until now, may justly give to the opinion, that *Cochituate water may be served from leaden pipes, connected with iron mains, without detriment to health.*' The opinion here expressed would command a high degree of confidence if it stood alone. Confirmed as it is by an evidence of collateral testimony, derived from long continued experience, we consider it entitled to entire confidence. The experiments detailed in Professor Horsford's first report, exhibiting the chemical action of the water of the Fairmount, Croton, and Jamaica Pond Water Works, and of the Cochituate lake, prove that there is a strong similarity in the effects of the waters from those several sources, upon lead.

"The ample testimony, founded on the continued use of the waters from the three first named sources, for a series of years, by thousands of families, without a single distinctly proved case of lead poisoning, although the water is served from the mains to the dwelling houses almost universally through leaden pipes, affords as satisfactory demonstration as the nature of the case admits of, that the Cochituate water may be safely distributed in the same way." p. 12.

Evidence, in regard to the absence of noxious lead impregnation from the New York, Philadelphia and other waters in which leaden service pipes have been and are employed, is given by numerous chemists and physicians; and the Report concludes as follows:—

"The grounds on which lead is preferred for the composition of small distribution pipes are, that the metal is cheap; it is easily formed into pipes, of any convenient size or length; it is flexible and easily adapted to all situations, in which it is desirable to place it; it is of sufficient strength to bear the pressure of any ordinary head of water, and if made of a suitable thickness, and provided with proper guards against the effects of a sudden check of the current, it is capable of resisting the extraordinary shock thus produced. It moreover preserves the water in a state of purity, and is itself durable, unless dissolved by the action of substances foreign to the source from which the city is to be supplied. Pipes of this material may be laid in a much shorter space of time, and at less cost, than those of cast iron.

"We have, therefore, on these considerations, resolved to use leaden pipes, for concduting the water to houses, except in cases in which the owners or occupants shall make known their preference of iron pipes, and announce their determination to make use of pipes of iron, or of some other material than lead, for the conveyance of the water through their respective houses, to the place of delivery, for use, for culinary purposes. Persons making such requests will be furnished with the water by means of pipes of cast iron.

"Having thus expressed our views in regard to the material of which the pipes should be composed, we proceed to comply with the order of the City Council, requesting our opinion 'as to the best and most economical mode of introducing water into private houses.'

"We recommend, that when leaden pipe is used, it should be five-eighths of an inch in diameter, weighing about three pounds to a foot in length, and that it be conducted through such part of the cellar, as will afford the best protection against frost, to the kitchen or sink-room, where the most constant supply will be required. As the water will rise, in most parts of the city, to any part of the house, in which it may be desired, and will be ready to flow from the pipe at all times with a rapid current, no tank or pump will be necessary. From any part of this pipe, a perpendicular branch may be carried to any other part of the house where the water may be desired, for the supply of baths, or any other purpose, care being taken to place it near the chimney, or in such position that it will be best protected against freezing. All pipes not so protected, should be laid with such an inclination, as will admit of their being emptied, when there is danger of freezing, by opening a discharge cock, to be placed at the lowest point. Pipes in being carried through coal cellars, and other exposed places, should be carefully protected against frost. It is desirable that the pipes shall be so placed as to be accessible for observation or repair.

"With these precautions, and by the employment of a skilful plumber to adjust the fixtures, the water may be conveyed to any part of the house at the pleasure of the occupant. Those who may choose to avoid the expense of such fixtures, may receive the water from a single stop-cock, at the place at which the pipe is introduced into the premises, or, which will be preferable, at the sink-room or kitchen. At all places of discharge there should be a sink, with a pipe to carry off the waste water. To every stop-cock also should be attached a piece of vacant pipe, or other air chamber above it which, by the compression of the air on the sudden shutting off of the water, may serve to relieve the pipe from the shock of what is called the *water hammer*. Otherwise, on account of the rapidity of the current, from the pressure of so high a head of water, as will rest on the pipes throughout the greater part of the city, they will be liable to be burst, or gradually expanded by repeated shocks." p. 22.

The Report is well worthy of being filed away by professional and municipal authorities; for the same question will doubtless recur when some other town is to be supplied with water. It is, as before said, ably drawn up; and the Appendix contains five excellent Reports of Professor Horsford; with Letters from John B. Jervis, Esq., Consulting Engineer of the Boston Water Works; from F. Boott, M. D., of London; from Professor Dunglison, not "of the University of Pennsylvania," as the Report inadvertedly states; from Dr. Benjamin H. Coates, and from Dr. Hare, of Philadelphia; from Dr. Lincoln, of Lancaster (Mass.); from Professor Hubbard, of Hanover, New Hampshire; from Dr. Thomas C. Brinsmade, of Troy; from Professor James McNaughton, of Albany; and from Professor Aikin, of Baltimore. From the letter of Mr. Jervis we make the following pregnant extract:—

"A trial in the U. S. Courts, as to patent of tinning lead pipe, in this city was had. On this trial, Prof. Renwick was called as a witness, and testified decidedly as to the injury of unprotected lead on water, and the necessity of protecting by tinning the lead;—that he would not, on any account, use lead pipe without tinning. On cross-examination—Do you use Croton water? Yes. Ever experience any injury? None whatever. What pipe is used? I presume tinned lead. The plumber who put in the pipe was then called, and testified that the pipe laid was all common lead pipe.

On the same trial Doct. Lee was called, who testified strongly as to the deleterious effect of lead pipe—that he would not have the water brought into his house, because his landlord refused to put in tinned pipe, and he therefore got his water in the street, from a free hydrant, where he said no lead was used. On cross-examination—Have you experienced any ill effects from using the Croton water from the street hydrant? No. The plumber who put in this hydrant was called, and testified that the pipe used for the hydrant was *pure lead*." p. 35.

